# Sarcomatoid urothelial carcinoma of the renal pelvis treated with immunotherapy

**DOI:** 10.1186/s12894-023-01210-z

**Published:** 2023-03-18

**Authors:** Tsutomu Anraku, Hideki Hashidate, Asa Nakahara, Tomoyuki Imai, Yoshiaki Kawakami

**Affiliations:** 1 Department of Urology, Niigata City General Hospital, 463-7 Shumoku, Chuo-ku, Niigata-City, Niigata 950–1197 Japan; 2 Department of Pathology, Niigata City General Hospital, Niigata, Japan

**Keywords:** Sarcomatoid, Urothelial carcinoma, Renal pelvis, Immunotherapy, Programmed cell death ligand 1, Cytotoxic T-lymphocyte antigen 4

## Abstract

**Background:**

Sarcomatoid carcinoma is a rare, high-grade malignancy with epithelial and mesenchymal components. It may be a good candidate for immunotherapy because it is associated with overexpression of programmed cell death ligand 1. Sarcomatoid urothelial carcinoma (UC) of the upper urinary tract is extremely rare. Here we report the first case of sarcomatoid UC of the renal pelvis that responded to immunotherapy.

**Case presentation:**

A 79-year-old man was referred to our hospital complaining of various symptoms, including anorexia and abdominal pain. A computed tomography scan revealed a right atrial tumor, a 9 cm left renal mass with a renal vein tumor thrombus, para-aortic lymphadenopathy, and multiple small lung nodules. The patient underwent resection of the right atrial tumor. Pathological analysis of the tumor did not lead to an accurate diagnosis even after several rounds of immunohistochemistry. He underwent a needle biopsy of the left kidney and was initially diagnosed with collecting duct carcinoma, a rare subtype of renal cell carcinoma (RCC). Following the initial diagnosis, immunotherapy with nivolumab and ipilimumab commenced. Thereafter, almost all lesions, including the left renal tumor, were reduced in size. However, he underwent a left nephrectomy approximately a year after beginning immunotherapy due to repeated left renal bleeding. Histological examination of the nephrectomy specimen revealed two forms of cancer—sarcomatoid UC and conventional high-grade UC. Two months after surgery, the patient was found to have new lung metastases. He underwent chemotherapy with gemcitabine and cisplatin, followed by immunotherapy with pembrolizumab. However, both treatments were ineffective. The patient died of cancer 19 months after his first admission.

**Conclusions:**

The presented case of sarcomatoid UC of the renal pelvis that partially responded to immunotherapy suggests that immunotherapy can be a promising treatment for sarcomatoid UC.

**Supplementary Information:**

The online version contains supplementary material available at 10.1186/s12894-023-01210-z.

## Background

Sarcomatoid carcinoma is a rare, high-grade malignant carcinoma that exhibits morphological and/or immunohistochemical evidence of epithelial and mesenchymal differentiation. The tumor can arise in many cancer types, including urothelial carcinoma (UC) in the urinary tract. Sarcomatoid UC is extremely rare and accounts for 0.3% of all urothelial cancers. Sarcomatoid UC often develops at an advanced stage and has a poor prognosis. Furthermore, sarcomatoid UC is chemoresistant, and to date, no benefit of conventional chemotherapy on overall survival has been confirmed [1, 2].

In recent years, significant progress has been made in immunotherapy for various cancer types. In particular, inhibition of the programmed cell death protein 1(PD-1)/programmed cell death ligand 1 (PD-L1) axis is of great importance in cancer immunotherapy. PD-1 and PD-L1 are immune checkpoint molecules that act as co-inhibitors to suppress T-cell responses. When PD-L1 on the surface of cancer cells binds to PD-1 on the surface of activated cytotoxic T-cells, T-cell activity is suppressed and cancer cells evade the immune response. Therefore, the PD-1/PD-L1 axis represents the resistance of tumor cells to cancer immunity, and cancers with high PD-L1 expression are considered good candidates for immunotherapy [3]. Because sarcomatoid carcinoma is associated with high PD-L1 expression in many cancer types [4–10], sarcomatoid UC may be a good candidate for immunotherapy targeting the PD-1/PD-L1 axis. In this report, we present the first case of sarcomatoid UC of the renal pelvis that partially responded to immunotherapy.

## Case presentation

**Fig. 1:**
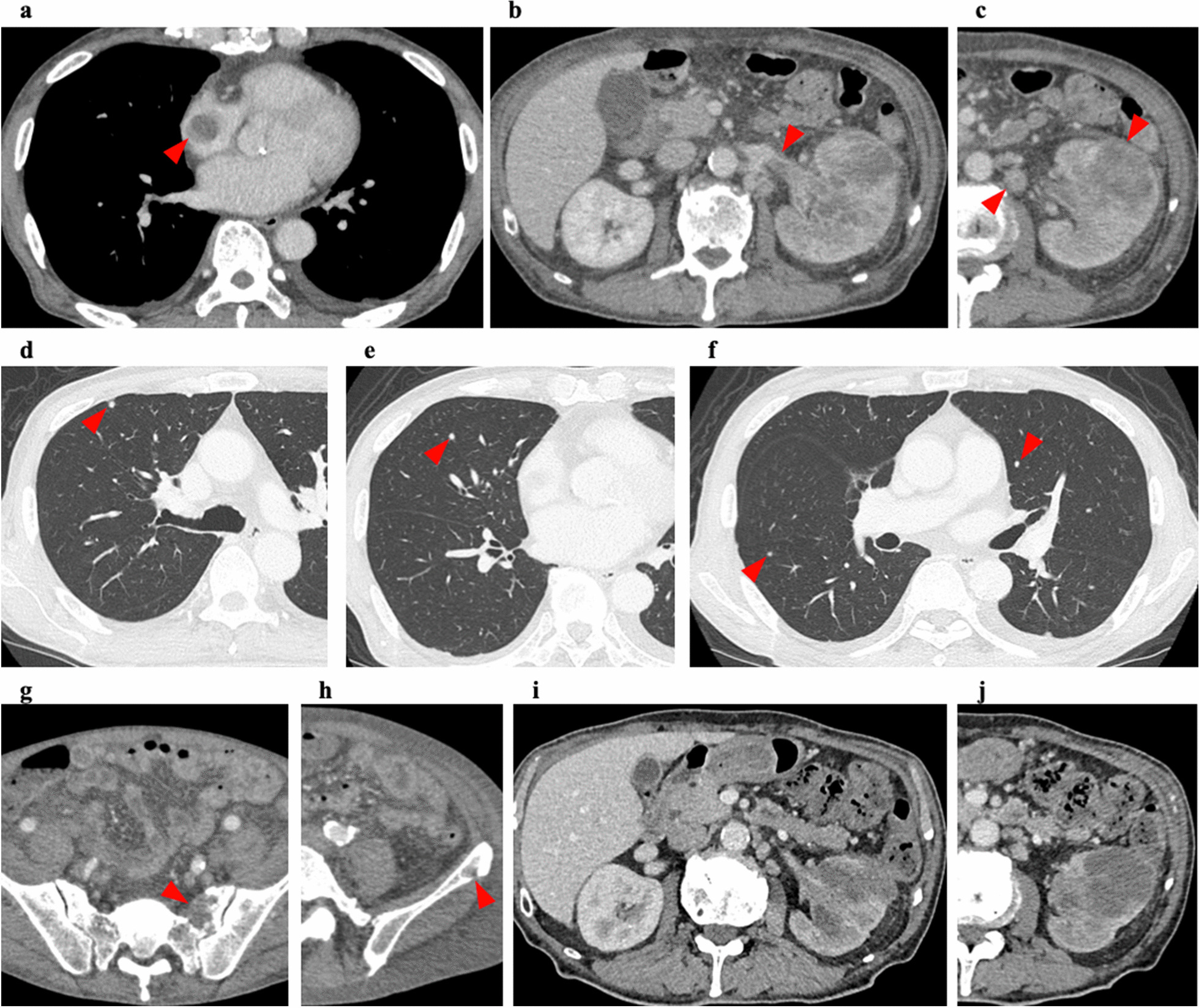
Chest and abdominal CT scan. Abnormal findings are indicated by arrowheads. **a** 18 mm mass found in the right atrium. **b** Tumor thrombus extending into the renal vein. The right kidney represents a normal and healthy control. **c** Heterogeneously enhanced left renal tumor. Para-aortic lymphadenopathy is also observed. **d**–**f** Multiple small lung nodules suspected of metastases. **g**, **h** Multiple bone metastases were observed after the second administration of ipilimumab and nivolumab. **i** Left renal vein tumor thrombus almost disappears following immunotherapy. **j** Left renal tumor and para-aortic lymphadenopathy remain reduced with continued immunotherapy

**Fig. 2:**
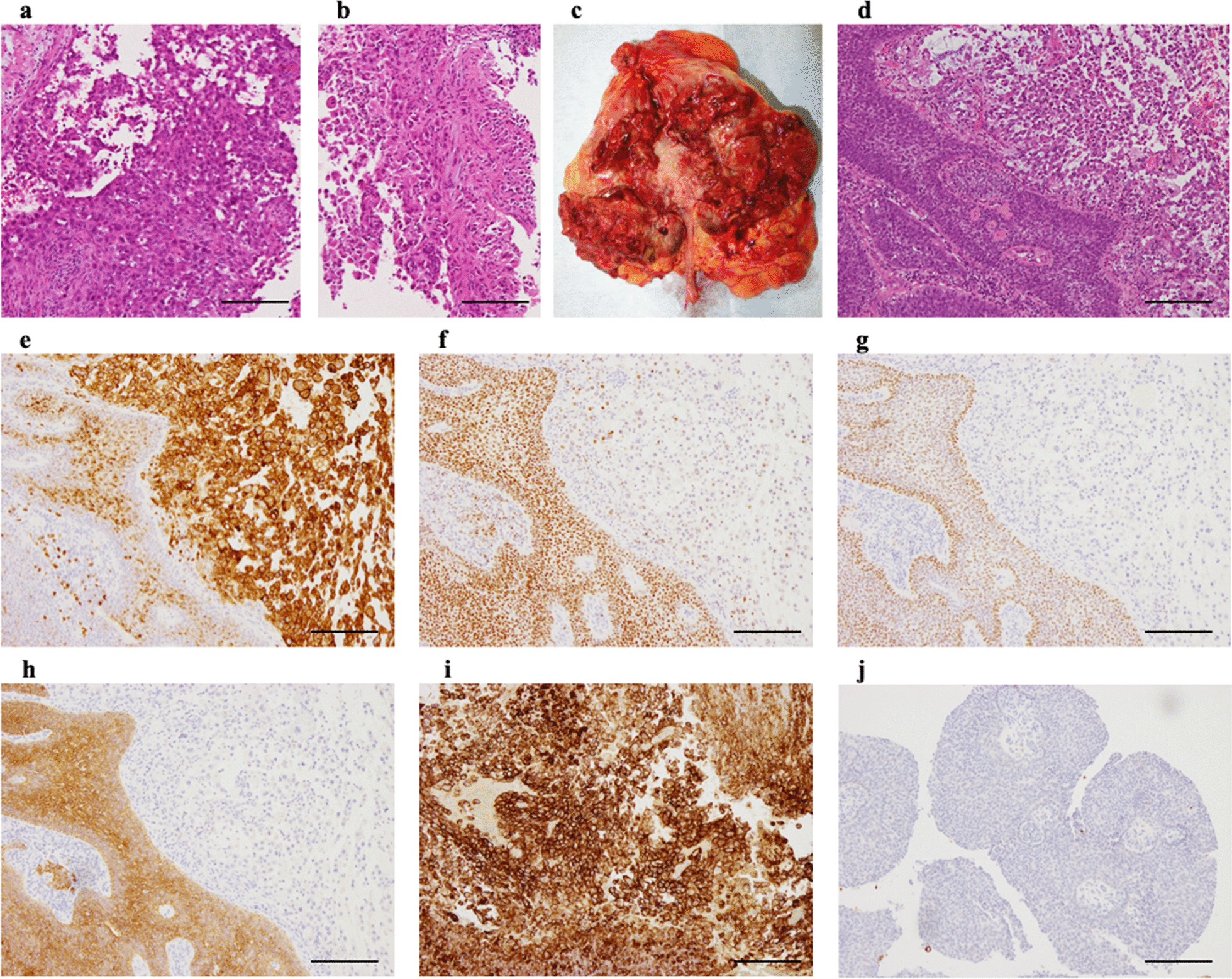
Pathological image of the specimens of right atrial mass, renal biopsy and left nephrectomy. **a** A microscopic examination of the atrial tumor shows metastatic carcinoma consisting of large atypical cells. **b** A microscopic examination of the needle biopsy of the left renal mass shows tumor tissue with high atypia and partial papillary growth. **c** Gross appearance of the nephrectomy specimen showing necrotic tumor infiltrating the renal parenchyma continuous with papillary tumors present in the renal pelvis. **d** Low-power images of hematoxylin and eosin staining showing both sarcomatoid and conventional UC components. The sarcomatoid component is composed of eosinophilic spindle-shaped cells. Immunostaining revealed that the sarcomatoid component is positive for CD10 (**e**), partially weakly positive for GATA-3 (**f**), and negative for p63 (**g**) and CK AE1/AE3 (**h**). PD-L1 is strongly expressed only in the sarcomatoid component (**i**) and not in the conventional UC component (**j**). The scale bar indicates 200 μm

In April 2020, a 79-year-old man with a medical history of hypertension and dyslipidemia was referred to Niigata City General Hospital (Niigata, Japan) after complaining of anorexia, weight loss, abdominal pain, and low-grade fever for approximately half a year. He had no family history of malignancy. Physical examination indicated mild emaciation. A blood test at the first visit revealed anemia (hemoglobin: 10.1 g/dl), leukocytosis (13,050/µl), and elevated C-reactive protein (3.46 mg/dl). Computed tomography (CT) scans revealed an 18 mm right atrial mass, a 9 cm left renal mass with a renal vein tumor thrombus, para-aortic lymphadenopathy, and multiple small lung nodules (Fig. 1a–f). Four days later, the patient underwent resection of the right atrial mass to prevent cardiac events associated with tumor embolism. Pathological examination showed metastatic carcinoma consisting of large, atypical cells attached to the endocardium and infiltrated the myocardium (Fig. 2a). However, the histology of the tumor could not be identified, even with various immunohistochemical examinations. Therefore, the patient underwent a needle biopsy of the left renal mass, which was thought to be the primary lesion, 11 days after surgery. Pathological examination of the needle biopsy specimen revealed a tumor tissue with high atypia and partial papillary growth (Fig. 2b). The immune profiles of atrial metastasis and renal biopsy specimens were as follows: positive for CK AE1/3, CK20, CK5/6, CK7, E-cadherin, GATA-3, PAX8, p63, vimentin, and 34βE12; negative for EMA, HMB45, KIT, and Melan-A; positive for CD10 in the atrial metastasis but negative in the renal biopsy specimen. Based on these results, the patient was diagnosed with collecting duct carcinoma (CDC). The clinical stage of the tumor was T3aN2M1, according to the Union for International Cancer Control 8th edition of the Tumor Node Metastasis Classification [11]. Immunotherapy with nivolumab (NIVO) and ipilimumab (IPI) was administered every three weeks at 240 mg and 1 mg/kg, respectively. After the second administration of NIVO and IPI, CT scans showed a mild reduction in all lesions noted in the previous CT scan. However, new bone metastases were observed (Fig. 1g, h). After four doses of NIVO and IPI, NIVO was administered at a dose of 240 mg every two weeks. A CT scan performed six months after the start of immunotherapy showed further reduction of all lesions, except for the bone metastases, which were unchanged. Another CT scan performed ten months after the start of immunotherapy showed that all lesions were stable (Fig. 1i, j). Following the CT scan, the patient experienced repeated bladder tamponade owing to left renal bleeding. Therefore, he underwent a left nephrectomy and para-aortic lymph node dissection.

**Fig. 3:**
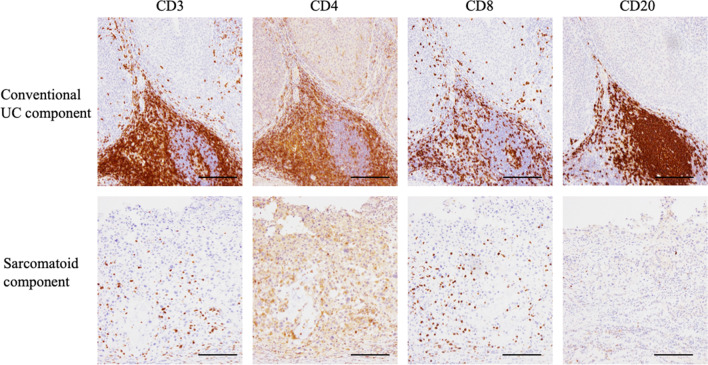
Immunostaining for lymphocytes in the nephrectomy specimen. Infiltration of CD8-positive T-cells into both sarcomatoid and conventional UC components of the tumor is observed. A significant accumulation of immune cells around the conventional UC components of the tumor is also seen. The scale bar indicates 200 μm

The nephrectomy specimen showed a 7.2 × 6.7 × 4.5 cm tan necrotic tumor, infiltrating the renal parenchyma with extension to the renal vein, continuous with papillary tumors present in the renal pelvis (Fig. 2c; Additional  file 1: Fig. S1). Histological examination revealed two forms of cancer: sarcomatoid UC infiltrating the renal parenchyma and high-grade UC in the renal pelvis (Fig. 2d). The sarcomatoid and conventional UC components were present at a ratio of approximately 70% and 30%, respectively. The sarcomatoid component of the tumor was composed of eosinophilic spindle-shaped cells with poor epithelial connections, extensive necrosis, and partial papillary growth. Immunohistochemistry revealed that the sarcomatoid and conventional UC components had completely different immune profiles. The sarcomatoid component was positive for CD10, weakly positive for CAM5.2, partially weakly positive for GATA-3, and negative for p63 and CK AE1/AE3 (Fig. 2e–h). PD-L1 was strongly expressed in the sarcomatoid component, but not in the conventional UC component (Fig. 2i, j). A small number of immune cells, mainly composed of CD8-positive T-cells, had infiltrated both the sarcomatoid and conventional UC components of the tumor. Besides, a significant accumulation of immune cells, including T cells and B cells, around the conventional UC components of the tumor was observed (Fig. 3). There were no metastases in the 11 removed lymph nodes. The final diagnosis was UC with sarcomatoid variant (pT3N0M1).

The patient was treated with denosumab for bone metastasis after surgery, and a CT scan performed two months later revealed newly emerging multiple lung metastases. Two cycles of chemotherapy with gemcitabine and cisplatin resulted in progressive disease. Pembrolizumab was administered at a dose of 200 mg every three weeks thereafter. However, a CT scan performed three months later revealed further progression of cancer, and the patient died 19 months after his first admission.

## Discussion and conclusions

Sarcomatoid carcinoma occurs as a result of sarcomatoid transformation caused by multiple stepwise gene mutations in cancer cells [12]. Several studies have shown that sarcomatoid transformation is associated with high PD-L1 expression in various cancer types, including UC [4–10]. PD-L1 is a biomarker for predicting the efficacy of immune checkpoint inhibitors; that is, cancers with high PD-L1 expression are more likely to respond to immunotherapy [13]. Kobayashi et al. reported that the presence of the sarcomatoid feature was significantly associated with better prognosis in a retrospective analysis of 755 patients with advanced UC who received immunotherapy [14]. Given that sarcomatoid UC is chemoresistant, immunotherapy is an attractive treatment option. Sarcomatoid carcinomas have also been reported to respond well to immunotherapy in other types of cancer, such as RCC and lung cancer [15, 16]. In addition, a preliminary study demonstrated the efficacy of immunotherapy in sarcomatoid mesothelioma [17].

We introduced immunotherapy with NIVO plus IPI for our case, whose actual diagnosis was sarcomatoid UC rather than CDC, a rare subtype of RCC. NIVO plus IPI has been approved by the United States Food and Drug Administration for various cancer types, such as RCC, melanoma, and lung cancer [18–23] but not for UC. Although the efficacy of NIVO plus IPI for UC has been shown to some extent, it is currently under further investigation in a clinical trial (NCT03036098) [24, 25].

The pathological diagnosis of sarcomatoid UC is difficult because it has a highly variable morphology and may resemble non-epithelial tumors [26]. It is even more difficult if the sarcomatoid UC component is predominant and the conventional UC component is absent. Differential diagnoses include carcinosarcoma, sarcomatoid RCC, primary sarcomas, and pseudosarcomatous myofibroblastic proliferation. Although immunohistochemistry is useful for differential diagnosis, staining characteristics overlap between these tumors [27]. In our case, cardiac metastasis and renal biopsy specimens, which were the bases for the initial diagnosis of CDC, were thought to be derived from the sarcomatoid component, which had a completely different histological morphology from conventional UC. PAX8 and GATA-3, sensitive markers of RCC and UC, were positive for immunostaining in our case, which is rare in sarcomatoid UC of the upper urinary tract. In contrast, PAX8 is usually positive and GATA-3 is positive on rare occasions in CDC [28, 29]. Considering that the conventional UC component was not observed in the needle biopsy specimens and the atypical immunohistochemical features of our case, in addition to the inherent difficulty of histological diagnosis of sarcomatoid UC, the renal needle biopsy could not provide an accurate diagnosis. The inaccurate initial diagnosis led the patient to receive immunotherapy with NIVO plus IPI, a non-standard treatment for UC, which was effective to some extent. If the patient was initially diagnosed with UC, he would receive cisplatin-based combination chemotherapy, which is the standard first-line treatment for metastatic UC but less effective for sarcomatoid UC. Since he was diagnosed with UC after the nephrectomy, he subsequently received chemotherapy. After the chemotherapy failed, we introduced immunotherapy with pembrolizumab, a second-line treatment recommended in this situation [30, 31]. The patient had some response to initial treatment with NIVO plus IPI, a dual blockade of PD-1 and cytotoxic T-lymphocyte antigen 4 (CTLA-4), but no response to the PD-1 antibody pembrolizumab. CTLA-4 is an immune checkpoint receptor that negatively regulates immune responses at the stage of T-cell priming. Therefore, CTLA-4 blockade has profound effects on particular T-cell populations important in anti-tumor immunity [32]. George et al. reported a case of sarcomatoid RCC that showed partial response to NIVO plus IPI and then progressed during maintenance therapy with NIVO [33], which is very similar to our case. The patient with sarcomatoid RCC was salvaged with the addition of IPI, which may suggest the importance of blocking CTLA-4 as well as PD-1 for sarcomatoid carcinoma.

Sarcomatoid UC often presents in an advanced stage, and systemic therapy is required in such cases. However, sarcomatoid UC is resistant to conventional chemotherapy, and a standard treatment protocol for sarcomatoid UC of the upper urinary tract has not yet been established because of the limited number of cases. To the best of our knowledge, only one case of sarcomatoid UC of the upper urinary tract treated with immunotherapy has been reported to date, and no effectiveness has been confirmed [34]. The patient in our case is the first reported case of sarcomatoid UC of the renal pelvis partially responded to immunotherapy, supporting the prospects of immunotherapy as a promising treatment for this disease. Further studies are required to investigate the effectiveness and optimal methods of immunotherapy for sarcomatoid UC of the upper urinary tract. Gene sequencing before starting immunotherapy may provide valuable information in determining drug efficacy.

In conclusion, we presented a case of sarcomatoid UC of the renal pelvis in which immunotherapy with NIVO plus IPI showed some effectiveness. The diagnosis of sarcomatoid UC is challenging, especially when the number of specimens is limited. Immunotherapy can be a promising treatment for sarcomatoid UC, and further investigation is needed to elucidate the effectiveness of immunotherapy for this tumor.

## Supplementary Information

**Additional file 1. Fig. S1**: Gross appearance of the nephrectomy specimen after formalin fixation.

## Data Availability

All data generated or analyzed during this study are included in this published article.

## Abbreviations

RCC: Renal cell carcinoma
PD-1: Programmed cell death protein 1
PD-L1: Programmed cell death ligand 1
CTLA-4: Cytotoxic T-lymphocyte antigen 4
UC: Urothelial carcinoma
CDC: Collecting duct carcinoma
