# Supplementary figures and images for: Sarcomatoid urothelial carcinoma of the renal pelvis treated with immunotherapy
Source: BMC Urol. 2023 Mar 18;23:38. doi: 10.1186/s12894-023-01210-z (PMC10024438; doi:10.1186/s12894-023-01210-z)

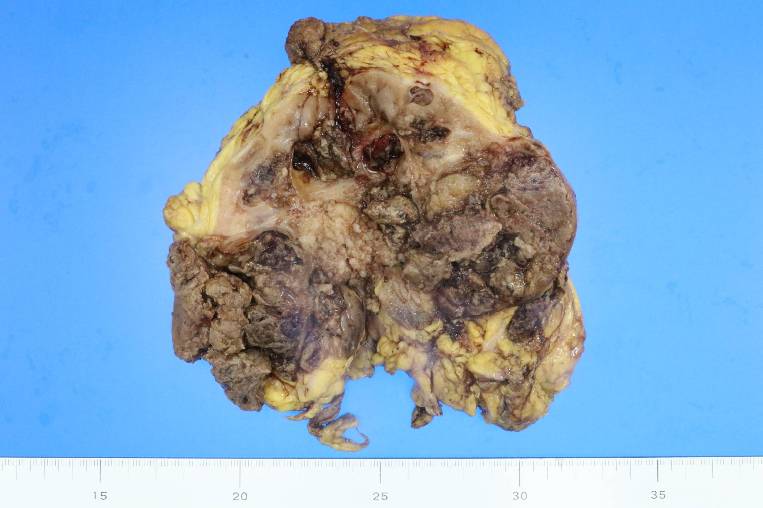

Supplement: Supplementary file 1 — Additional file 1. Fig. S1: Gross appearance of the nephrectomy specimen after formalin fixation. [file 12894_2023_1210_MOESM1_ESM.jpg]
